# 289 The Timing of Pharmacologic Venous Thromboembolism Prophylaxis Initiation for Burn Patients with Concomitant Trauma

**DOI:** 10.1093/jbcr/irad045.264

**Published:** 2023-08-29

**Authors:** Lourdes Castanon, Tanya Anand, Sai Krishna Bhogadi, Christina Colosimo, Michael Ditillo, Khaled El-Qawaqzeh, Lynn Gries, Hamidreza Hosseinpour, Bellal Joseph, Adam Nelson, Audrey Spencer

**Affiliations:** The University of Arizona, Tucson, Arizona; The University of Arizona, Tucson, Arizona; The University of Arizona, Tucson, Arizona; The University of Arizona, Tucson, Arizona; The University of Arizona, Tucson, Arizona; The University of Arizona, Tucson, Arizona; The University of Arizona, Tucson, Arizona; The University of Arizona, Tucson, Arizona; The University of Arizona, Tucson, Arizona; The University of Arizona, Tucson, Arizona; The University of Arizona, Tucson, Arizona

## Abstract

**Introduction:**

Hospitalized burn patients are at increased risk for venous thromboembolism (VTE). Clinical guidelines regarding thromboprophylaxis in burn patients with concomitant trauma are unclear. We aimed to compare the outcomes of early versus late thromboprophylaxis initiation for burn patients with concomitant trauma.

**Methods:**

A 3-year analysis of 2017-2019 TQIP. We identified all adult burn patients with concomitant trauma who received thromboprophylaxis. We excluded patients that had severe TBI (Head AIS > 2), were transferred, those that died < 24 hours of admission and those that underwent hemorrhage control surgery. Patients were stratified based on the timing of initiation of postoperative thromboprophylaxis: Early (< 24 hours of admission) versus Late ( >24 hours). Multivariate regression analyses were performed. Outcome measures were odds of deep venous thrombosis (DVT), pulmonary embolism (PE), unplanned return to OR, unplanned ICU admission, post-prophylaxis PRBC transfusion and in-hospital mortality.

**Results:**

9,272 trauma patients with concomitant burns who received thromboprophylaxis were identified. Overall, mean age was 41.3 ± 13.4 years, 71.5% were male, and median ISS was 3 [1-8]. 53% had second degree burns, and 39% had more than 10% of total body surface area affected. Median time to thromboprophylaxis initiation was 11 [6-20.6] hours. On multivariate regression, patients who received thromboprophylaxis after 24 hours of admission had 1.8 times higher odds of developing DVT (aOR=1.8, 95% CI=1.04-3.11, *p=*0.03), 4.8 times higher odds of developing PE (aOR=4.8, 95% CI=1.9-11.9, *p<*.001), 2 times higher odds of unplanned ICU admission (aOR=2.1, 95% CI=1.4-3.1, *p<*0.001), and nonsignificant increase in the odds of unplanned return to OR (aOR=2, 95% CI=0.96-4.31, *p=*0.06). Further, early thromboprophylaxis was not associated with increased odds of post-prophylaxis PRBC transfusion (aOR=1.1, 95% CI=0.8-1.4, *p=*0.4), and in-hospital mortality (aOR=0.68, 95%CI=0.4-1.1, *p*=0.13) compared to patients who received late thromboprophylaxis.

**Conclusions:**

Early VTE prophylaxis in burn patients is associated with decreased rates of DVT and PE, without increasing the risk of bleeding and mortality. VTE prophylaxis may be initiated within 24 hours of admission to reduce VTE in this high-risk patient population.

**Applicability of Research to Practice:**

This work will guide the timing of initiation of venous thromboembolism prophylaxis in burn patients with concomitant trauma

